# Entre innovación y marginalidad: la psiquiatría en el Rosario de entreguerras

**DOI:** 10.1590/S0104-59702025000100036

**Published:** 2025-09-15

**Authors:** Anatole Le Bras

**Affiliations:** i Profesor titular, Université de Versailles Saint-Quentin-en-Yvelines. Guyancourt – Francia anatole.lebras@sciencespo.fr

El libro *De la cárcel al hospital*, basado en la tesis doctoral de José Ignacio Allevi, investigador asistente en el Consejo Nacional de Investigaciones Científicas y Técnicas, nos invita a adentrarnos en el entorno psiquiátrico de una ciudad argentina en el periodo de entreguerras. Junto con otros estudios recientes de escala local o regional (Ferrari, 2016), este libro se suma a los relatos más generales sobre la historia de la psiquiatría en Argentina, a menudo enfocados en Buenos Aires (Balbo, 1991; Ablard, 2008). Ambiciona estudiar la constitución de un campo psiquiátrico, en el sentido bourdieuniano, y “el conjunto de factores que posibilitaron [la] circulación y aplicación [de los saberes psi]” (Allevi, 2021, p.35). El autor se interesa tanto por las instituciones y por las estrategias de legitimación disciplinar como por las prácticas clínicas, principalmente a la luz de fuentes éditas.

El libro de José Ignacio Allevi logra la proeza de mostrar que un lugar relativamente marginal de la psiquiatría argentina puede enseñarnos mucho sobre la historia de la psiquiatría y sus vínculos con la sociedad. Su relato nos muestra cómo se articulan los factores políticos y económicos y las cuestiones de fronteras disciplinarias.

Para empezar, el libro demuestra que la emergencia del campo “psi” en Rosario tuvo lugar en un contexto económico, social y político particular. Al principio del siglo XX, Rosario experimentó un desarrollo muy rápido, impulsado por su dinamismo económico y su situación de segundo puerto del país. Aunque la ciudad tenía poco poder político y administrativo por no ser capital provincial, existía una tradición de intervencionismo sanitario municipal. La estructuración de la Facultad de Medicina por el médico tucumano Antonio Agudo Ávila resultó de negociaciones entre el nivel nacional y la elite rosarina. La creación del hospital de Alienados de Rosario, estimulado por los avances de la nueva corriente de la higiene mental representada en Rosario por Lanfranco Ciampi, psiquiatra italiano y discípulo de Sante de Sanctis, llegó a término en octubre de 1927.

El autor también expone que el desarrollo de la psiquiatría en Rosario fue un proceso conflictual, marcado por una competencia entre el enfoque neurológico de Teodoro Fracassi, un médico muy influyente en Rosario, y los psiquiatras Ciampi y Raimundo Bosch. La creación del Instituto de Psiquiatría de la Universidad, en 1929, dando razón a Bosch y Ciampi, marcó la independencia de la psiquiatría con respecto a la neurología y permitió el desarrollo de la psicología experimental en Rosario.

Allevi reconstituye cuidadosamente las redes y los enlaces que modelaron al entorno psiquiátrico rosarino y permitieron su expansión. Los psiquiatras rosarinos estaban vinculados a las redes de la higiene mental, del eugenismo, e hicieron incursiones en el campo pedagógico – la apertura de una escuela de niños retardados en 1922, junto con una cátedra en psiquiatría infantil, manifestó el temprano interés de los psiquiatras rosarinos por el ámbito de la infancia. Finalmente, la psiquiatría rosarina se integró a las iniciativas de salud pública en los años 1930, y la experticia técnica del Instituto de Psiquiatría fue progresivamente reconocida por el Estado y por actores del sector educativo.

Sin embargo, las “interferencias políticas” (Allevi, 2021, p.94) y las consecuencias del golpe de Estado de 1930 amenazaron el fortalecimiento de la disciplina psiquiátrica. Además, la permanente escasez presupuestaria limitó seriamente las realizaciones de la profesión.

Así, *De la cárcel al hospital* nos da las claves para comprender cómo la psiquiatría se convirtió en una disciplina ambiciosa y versátil, pero siempre frágil.

Uno de los puntos fuertes del libro es la forma en que pone de relieve la inscripción de la psiquiatría rosarina en las redes internacionales y sus vínculos con Europa. Allevi pone exitosamente en práctica un juego de escalas, de lo local a lo global, gracias a un excelente dominio tanto de las fuentes locales como de la bibliografía internacional, de manera ejemplar en el cuarto capítulo. Este capítulo cuenta cómo el Instituto de Psiquiatría de Rosario contribuyó a la difusión internacional de la terapia convulsiva con cardiozol, iniciada por el húngaro Laszlo von Meduna. Bajo la influencia de su nuevo director Juan Cuatrecasas, el instituto incorporó cada vez más los aportes de la farmacología y de la endocrinología. Así, la institución rosarina se transformó “en un laboratorio antropológico que generaba su propio aporte a las teorías europeas que revolucionaban epistemológicamente la disciplina” (Allevi, 2021, p.165). A través del estudio de caso de las terapias de shock, como lo destaca Mariano Ben Plotkin en el prólogo, Allevi nos invita a repensar la relación centro-periferia, tanto al nivel argentino como al nivel internacional. Asimismo, llama a la reflexión sobre las condiciones particulares que vieron nacer a la primera cátedra de psiquiatría infantil del mundo en Rosario, quince años antes del primer congreso mundial de la disciplina en Paris (Boussion, Coffin, 2016).

Nos parece, sin embargo, que el estudio de Allevi tiene algunos puntos ciegos. En el relato propuesto por el autor, no se tienen en cuenta las prácticas de internamiento. Las cifras relativas a la internación de pacientes en el hospital sólo se mencionan de pasada. Poco se dice sobre el origen social y geográfico de los pacientes, el papel de las familias en el sistema de asistencia a los enfermos mentales, o las consecuencias concretas de la sobrepoblación institucional. No obstante, la capacidad de la psiquiatría para responder a una “demanda social” de reclusión o, por el contrario, el cuestionamiento de su dimensión “carcelaria”, son dimensiones importantes de la construcción de su legitimidad social (Porter, Wright, 2003). Un uso más profundo de la prensa también podría haber aportado elementos interesantes, dado que la construcción de la autoridad social de los psiquiatras no sólo se juega en su relación con las autoridades públicas o con sus colegas médicos, sino también en su relación con la población y la opinión pública.

Estas observaciones no restan valor al libro, que puede ser de interés para una amplia audiencia, mucho más allá de los historiadores de la salud en Argentina.
